# Syndrome of Inappropriate Anti‐Diuretic Hormone Secretion With Hyponatremia in Viral Encephalitis Patient: A Case Report

**DOI:** 10.1002/ccr3.71322

**Published:** 2025-10-16

**Authors:** Juan Fu, Yan Zhao, Shuyi Li, Li Ren, Qingsong Wang, Binfeng He, Zaichun You

**Affiliations:** ^1^ Department of General Practice Xinqiao Hospital, Third Military Medical University (Army Medical University) Chongqing China

**Keywords:** case report, hyponatriemia, syndrome of inappropriate antidiuresis, viral encephalitis, viral infection

## Abstract

The syndrome of inappropriate antidiuretic hormone secretion is a condition marked by the excessive production of antidiuretic hormone, potentially resulting in hyponatremia. If not properly managed, severe hyponatremia can result in seizures, cerebral edema, and even death. There are many causes of this inappropriate release of antidiuretic hormone, including malignant tumors, central nervous system infections, drug‐induced factors, and hypothalamic–pituitary–renal axis disorders. Current evidence suggests that viral encephalitis‐related SIADH manifestations are markedly underrepresented in existing medical reports. We reported a case of elderly SIADH with persistent dizziness and fatigue as the main symptoms. Through multidisciplinary comprehensive analysis and discussion, and after excluding various factors, we considered it to be caused by viral encephalitis, which is rare in clinical practice. The SIADH is a clinically challenging disease that can severely impact a patient's health and quality of life. General practitioners need to consider multiple factors when diagnosing and treating this condition, leveraging their expertise, collaborating with multidisciplinary teams, and focusing on early identification and accurate diagnosis. They should develop individualized treatment plans and implement comprehensive management strategies to ultimately cure the patient.

**Summary**

- We present a challenging case of an elderly patient with SIADH secondary to viral encephalitis, presenting with recurrent dizziness and fatigue.
- After extensive diagnostic exclusion and empirical antiviral therapy, the patient recovered fully.
- Two‐year follow‐up confirmed sustained remission, highlighting the importance of thorough evaluation in atypical presentations.

## 1 Introduction

SIADH is a condition characterized by hypotonic and euvolemic hyponatremia due to impaired free water excretion resulting from the release of anti‐diuretic hormone without adequate stimuli [1]. Hypotonic, normal blood volume hyponatremia, and urinary hyperosmotic pressure are signs of it. It is induced by malignant tumors, drugs (such as antidepressants, tricyclic drugs, monoamine oxidase inhibitors), infection (such as lung abscesses, tuberculosis, pulmonary aspergillus, brain abscesses, encephalitis, meningitis), neurological disorders (epidural hematoma, subarachnoid hemorrhage, brain tumors, head trauma) [1, 2]. Hypotonic hyponatremia is a common symptom of SIADH, and if left untreated, severe hyponatremia can lead to seizures, cerebral edema, and potentially fatal outcomes [3].

In the literature, there are a few cases of symptomatic hyponatremia caused by SIADH due to viral encephalitis. Adeoluwa Ayoola et al. [4] present a clinically significant case of syndrome of inappropriate antidiuretic hormone secretion (SIADH)‐induced symptomatic hyponatremia in a patient with prostatic adenocarcinoma, occurring as a postprocedural complication following high‐dose‐rate (HDR) brachytherapy administered under general anesthesia. Matthew Smale et al. [5] presented a case of severe hyponatremia caused by SIADH, which occurred in the context of human respiratory syncytial virus infection.

The present report describes a case of SIADH that resulted in hyponatremia in a patient who had a viral infection. SIADH has not been previously documented in adults with viral infections, as far as we know. This patient provided written informed consent.

## 2 Case History/Examination

The subject, a 66‐year‐old female, was brought into Xinqiao Hospital on March 12, 2022, experiencing recurrent dizziness and fatigue that persisted for 20 days and were aggravated due to cough and sputum production over the prior 3 days. Her initial symptoms had been dizziness, generalized muscle pain, and a mild fever with a peak body temperature of 37.9°C. She initially experienced dizziness along with generalized muscle pain and a low‐grade fever, peaking at a maximum body temperature of 37.9°C, approximately 20 days ago. There were no indications of altered consciousness, headache, nausea, or vomiting. She went to the nearby outpatient clinic for medical consultation and was diagnosed with a viral infection. The patient received a Chinese patent medicine known for its antiviral particles (active ingredients of Antiviral Granules include (R, S)‐goitrin, indigo, indirubin, salicylic acid, benzoic acid, and adenosine), at a dosage of 9 g per day for 5 days. Regrettably, the treatment did not yield significant improvement in her symptoms, and she experienced aggravated dizziness, along with symptoms of nausea, vomiting, reduced appetite, and fatigue. She came to the Xinqiao Hospital Emergency Department on February 26, 2022, reporting dizziness, fatigue, nausea, and vomiting. Blood analysis revealed a potassium level of 2.9 mmol/L and a sodium level of 130 mmol/L. Symptoms of fatigue, nausea, and vomiting improved after they administered 10 mg metoclopramide and a 1.5 g potassium chloride injection; still, her dizziness endured. Despite an ensuing treatment of 0.5 g Ribavirin injection for 7 days at the local outpatient clinic starting from March 2, 2022, her dizziness didn't significantly improve, and by March 9, 2022, her dizziness and fatigue had worsened. Furthermore, she also suffered from hand tremors, cough, and white, frothy, hard‐to‐expectorate sputum.

## 3 Other Related Medical History

The patient has been residing in the Shapingba District of Chongqing and has no history of exposure to epidemic areas or contaminated water. She has received the SARS‐CoV‐2 vaccine, and the result of her SARS‐CoV‐2 and respiratory viral panel (RVP) test at our hospital was negative (see Table 1). The patient denies having any history of malignancy, epilepsy, tuberculosis or psychiatric illnesses, and has not taken any medication associated with these conditions.

**TABLE 1:** The results of the patient's respiratory viral panel (RVP) test.

	Item	Result
Respiratory pathogen nucleic acid	Adenovirus	Negative
	Rhinovirus	Negative
	Respiratory Syncytial Virus (RSV)	Negative
	Influenza A Virus	Negative
	Influenza B Virus	Negative
	SARS‐CoV‐2	Negative

## 4 Investigations and Treatment

**FIGURE 1:**
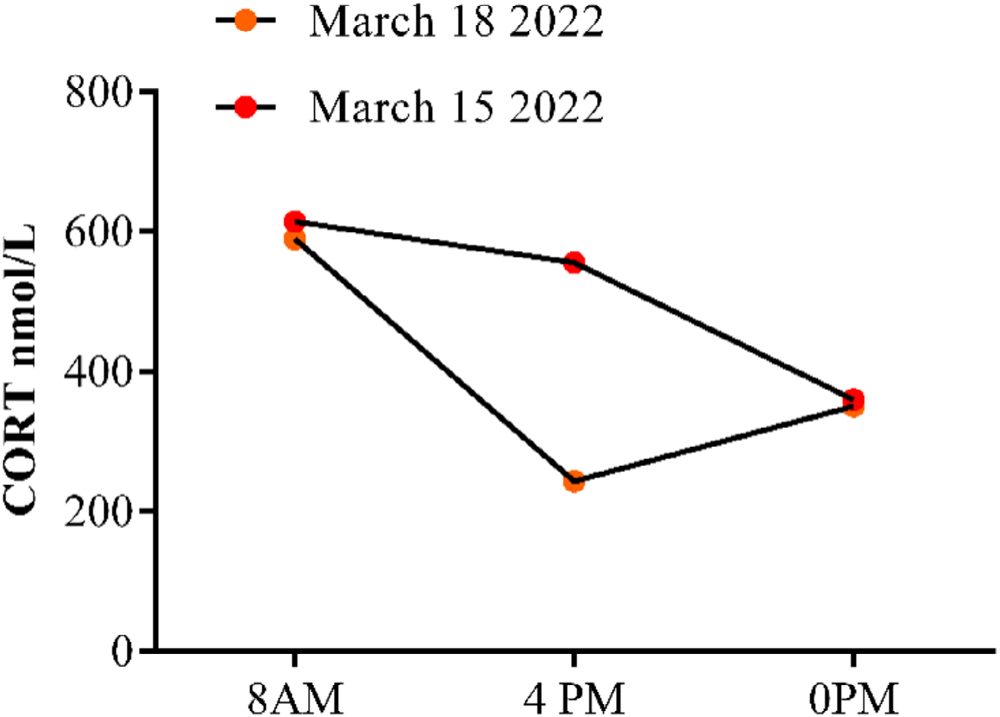
The changes in cortisol rhythm after illness (patient context).

During the initial hospitalization, she received levofloxacin 0.5 g for anti‐infection therapy excluding coronavirus, tuberculosis, and other reasons induced lung infection, daily sodium supplementation of 16–18 g, potassium supplementation of 4–6 g, and parenteral nutrition support among other measures for 5 days, which dramatically improved her symptoms of cough, sputum, and diarrhea, but dizziness, fatigue, and low blood sodium levels persisted. Sodium excretion was recorded at 453.72 mmol/24 h, with blood osmotic pressures of 248 mOsm/Kg and urine osmotic pressure of 392 mOsm/kg during a 24‐h urine collection (Table 2). Her renin‐angiotensin system activity (Table 3), cortisol rhythm (Table 4, Figure 1), sex hormones, 17‐hydroxycorticosteroid levels, brain natriuretic peptide, CMV, VZV/HSV, EBV, RV, TOX (Table 5), thyroid function test, adrenal CT, and pituitary MRI were normal (Table 6). Consequently, the patient has been contemplated for SIADH.

**TABLE 2:** The value of hematuria osmotic pressure before and after treatment in patients.

Date	Blood osmotic pressure (mOsm/kg)	Urine osmotic pressure (mOsm/kg)
March 18, 2022 (Before treatment)	248↓	392↓
April 02, 2022 (Posttreatment)	278	610

**TABLE 3:** Patient's renin‐angiotensin system test results.

	Ang I (37°C)	Ang I (0°C)	Ang II (pg/mL)	PRA (ng/mL/h)	CORT (nmol/L)	ALD‐W (pg/mL)
Clinostatism	0.1	0.09	78.64	0.01	402	30.8
Erect position	0.58	0.2	79.06	0.38	785.1	146.1

Abbreviations: ALD‐W, aldosterone; Ang I, angiotensin I; Ang II, angiotensin II; PRA, plasma renin activity.

**TABLE 4:** Patient's CORT and ACTH test results.

	CORT (8AM) (nmol/L)	CORT (4PM) (nmol/L)	CORT (0PM) (nmol/L)	ACTH (ng/L)
March 15, 2022	614.3	555.9	359.5	38.36
March 18, 2022	589.4	243.3	350.5	50.65

Abbreviations: ACTH, adrenocorticotropic hormone; CORT, cortisol.

**TABLE 5:** The results of the patient's TORCH panel and Epstein–Barr virus test.

	Item	Result
TROCH	Toxoplasma gondii	Negative
	Varicella‐Zoster Virus (VZV)	Negative
	Rubella virus	Negative
	Cytomegalovirus (CMV)	Negative
	Herpes simplex virus (HSV)	Negative
EBV	Epstein–Barr virus (EBV)	Negative

**TABLE 6:** Important imaging results of patients.

Inspection Items	Result
Chest CT (March 14, 2022)	Tiny indurations in the apical segment of the upper lobe of the right lung and the upper lingual segment of the upper lobe of the left lung may be
Brain CT (March 14, 2022)	There was no obvious abnormality in brain parenchyma
CE‐pituitary MRI (March 17, 2022)	No abnormal signal shadow was found in the saddle area
CE‐adrenal CT (March 18, 2022)	There was no obvious abnormality in adrenal CT plain scan and enhancement
CE‐MRI (March 20,2022)	There was no abnormal enhancement in brain parenchyma
PET/CT (March 29, 2022)	1. PET/CT imaging of the head and body showed no obvious tumor signs 2. There was no abnormal increase in FDG metabolism in the apical segment of the upper lobe of the right lung and the lingual segment of the upper lobe of the left lung. The possibility of induration was considered and followed up
EEG (April 01, 2022)	There was no obvious abnormality in electroencephalogram

Subsequently, her condition, marked by persistent symptoms of dizziness and fatigue, often associated with SIADH, also indicative of cranial injuries and brain tumors, was extensively examined. Cerebrospinal fluid analyses revealed positive Pan's tests, an elevated count of white blood cells—primarily monocytes, increased protein content, and reduced chloride ion count (Table 7). However, no considerable anomalies were observed in cerebrospinal fluid NGS tests (Table 7), head CT, head MRI, or PET‐CT (Table 6). A munificent treatment approach was devised, limiting her daily fluid intake to no more than 800 mL, prescribing sodium supplementation (13 to 16 g/day), initiating antiviral therapy with acyclovir (0.5 g q8h), and availing psychological counseling.

**TABLE 7:** Routine and biochemistry analysis of cerebrospinal fluid.

	PAN	WBC (10^6^/L)	Protein (g/L)	PMNCs (%)	MONO (%)	GLU (mmol/L)	Cl^−^ (mmol/L)	Pressure (mmH_2_O)	mNGS
March 17, 2022	Positive	82↑	0.77↑	1	99	3.69	111↓	99	Negative
March 23, 2022	Positive	92↑	0.72↑	2	98	2.96	114.8↓	135	Negative

Abbreviations: Cl^−^, chloride; GLU, glucose; MNC_S_, polymorphonuclear cells; mNGS, metagenomic next‐generation sequencing; MONO, mononuclear cells; PAN, Pandy's test; WBC, white blood cell.

## 5 Conclusion and Results (Outcome and Follow‐Up)

**FIGURE 2:**
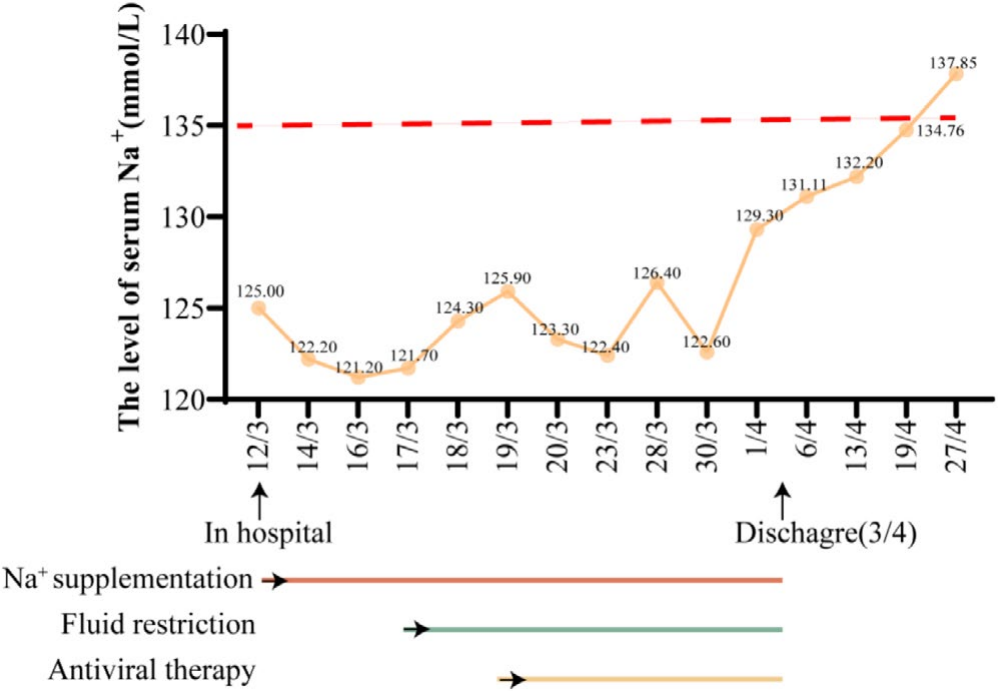
The changes in sodium ion levels during the patient's hospitalization and after discharge.

After 5 days of treatment, her dizziness and fatigue significantly improved, deeming her fit for discharge on April 2, 2022. The serum sodium was 129.3 mmol/L, and the blood osmotic pressure and urine osmotic pressure returned to normal (Table 2). Post‐discharge weekly follow‐ups incorporated observing her blood sodium levels and noting the gradual diminution of her dizziness and fatigue symptoms. Sodium levels entirely normalized after 4 weeks (Figure 2 and Table 8). Telephonic follow‐ups conducted 6 months and 1 year post‐discharge indicated a favorable condition and promising prognosis, thereby supporting the diagnosis of SIADH attributed to viral‐induced brain infection.

**TABLE 8:** Changes in electrolyte levels in patients.

	12/3	14/3	16/3	17/3	18/3	19/3	20/3	23/3	28/3	30/3	1/4	6/4	13/4	19/4	27/4
Na^+^ (mmol/L)	125.0↓	122.2↓	121.2↓	121.7↓	124.3↓	125.9↓	123.3↓	122.4↓	126.4↓	122.6↓	129.3↓	131.11↓	132.2↓	134.76	137.85
Cl^−^ (mmol/L)	91.7↓	89.9↓	90.5↓	88.8↓	93.0↓	93.6↓	86.8↓	88.8↓	89.8↓	85.5↓	92.6↓	88.22↓	93.67↓	95.36↓	98.19
K^+^ (mmol/L)	4.07	3.29↓	3.69	3.29↓	3.35↓	3.48↓	3.86	4.21	4.00	3.75	4.26	3.75	3.64	4.13	4.54

## 6 Discussion

This case report delineates an instance of hypotonic hyponatremia attributed to SIADH, determined after eliminating other possible causes. The diagnosis of SIADH, associated with a viral brain infection, was substantiated based on the observed symptoms of dizziness and the introduction of an antiviral regimen.

Certain malignant tumors, respiratory conditions (like pneumonia), central nervous system diseases, and viral infections (for instance RSV, EBV, COVID‐19) have been identified as significant factors contributing to SIADH development with hyponatremia [5, 6, 7, 8, 9, 10]. However, diagnosing the etiology of SIADH can prove immensely challenging, particularly in elderly patients suffering from chronic diseases, who tend to exhibit a range of nonspecific symptoms and abnormal biochemical parameters.

The patient presented with persistent hyponatremia, difficulty in sodium supplementation, decreased plasma osmotic pressure (248 mOsm/kg), inappropriate urinary osmotic pressure (392 mOsm/kg), increased 24‐h urinary sodium excretion (453.72 mmol/h), and normal blood volume. After the exclusion of hypothyroidism and adrenocortical insufficiency, the diagnosis of SIADH was considered. A comprehensive assessment is needed to identify potential causes. The diagnostic process focused on excluding several key diseases. (1) Hypovolemic or hypervolemic hyponatremia: adrenal insufficiency and hypothyroidism were excluded by cortisol, ACTH, thyroid function tests, and adrenal CT, because these conditions usually exhibit different hormonal characteristics and are poorly responsive to individual sodium correction. Renal salt depletion is considered impossible because there is no use of diuretics. (2) Nervous system lesions: head imaging (CT and MRI) excludes ischemic stroke, intracranial space‐occupying lesions, or traumatic injuries. However, the abnormal detection results of cerebrospinal fluid cannot exclude viral encephalitis or autoimmune encephalitis. (3) Cerebral salt‐wasting syndrome (CSWS) needs to be considered but can be ruled out due to adequate blood volume and no history of intracranial hypertension or neurosurgery. (4) SIADH‐related etiologies: drug‐induced SIADH: a comprehensive drug review confirmed that the patient had not been exposed to antidepressants, antipsychotics, or chemotherapeutic agents known to induce SIADH. Tumor etiology: Whole‐body positron emission computed tomography (PET/CT) ruled out paraneoplastic SIADH (e.g., small‐cell lung cancer), and no occult malignancies were identified. Causes of infection: chest CT, head‐enhanced MRI, tuberculosis‐related tests, sputum culture identification, cerebrospinal fluid culture identification, and cerebrospinal fluid NGS did not reveal evidence of (bacterial/fungal pneumonia, tuberculosis, and lung abscess) lung infection or (meningitis, encephalitis, and brain abscess) central nervous system infection.

After ruling out the common diseases causing SIADH as described above, the combination of the patient's persistent symptoms of dizziness and the fact that both cerebrospinal fluid examinations suggested abnormal findings in the cerebrospinal fluid, predominantly elevated mononuclear cells, elevated albumin, and lowered chloride ions in the cerebrospinal fluid. And the possible presence of actual CNS infection or encephalopathy mediated by postinfectious secondary immune activation, which may not be demonstrated by MRI. In addition, CNS infections may present neurologic dysfunction and endocrine dysfunction [11]. We believe that SIADH was associated with intracranial viral infection in our patient. Therefore, we decided to continue full antiviral therapy with acyclovir, which resulted in significant symptomatic relief and subsequent successful remission of SIADH and no dysfunction. Notably, one limitation of our case was the inability to identify the virus, implying the causality of SIADH could only be inferred but not conclusively proven.

Hyponatremia, a heterogeneous disorder, can be triggered by SIADH, lung disease, hypovolemia, and heart failure [12]. However, physicians often overlook hyponatremia, particularly in elderly patients or those affected by chronic diseases, who may present various symptoms during preliminary evaluations. Insufficient management in such cases may lead to less‐than‐ideal patient outcomes. In this case, we confirmed hyponatremia stemming from SIADH, following which we implemented a treatment approach comprising fluid restriction, sodium supplementation [13], and the continuation of the antiviral protocol. The patient's serum sodium levels eventually normalized, leading to an improvement in symptoms.

## 7 Conclusion

In summary, we detailed a case showcasing SIADH‐induced hyponatremia prompted by a brain infection in an elderly patient. This case underlines the significance of considering SIADH as a potential cause for hyponatremia in patients, leveraging the comprehensive capabilities of general practice and promoting collaboration among diverse healthcare professionals.

## Author Contributions

**Juan Fu:** conceptualization, investigation, writing – original draft. **Yan Zhao:** data curation. **Shuyi Li:** data curation. **Li Ren:** data curation. **Qingsong Wang:** data curation. **Binfeng He:** conceptualization, writing – original draft, writing – review and editing. **Zaichun You:** conceptualization, data curation, writing – review and editing.

## Ethics Statement

Informed consent was obtained from the individuals for the publication of any potentially identifiable images or data included in this article.

## Conflicts of Interest

The authors declare no conflicts of interest.

## Data Availability

The data that support the findings of this study are available from the corresponding author upon reasonable request.
